# Case series: Intraoperative neuromonitoring and angiography in the surgical treatment of vascular malformations

**DOI:** 10.3389/fneur.2023.1182576

**Published:** 2023-10-26

**Authors:** Tomaž Šmigoc, Ninna Kozorog, Janez Ravnik

**Affiliations:** Department of Neurosurgery, Surgical Clinic, UMC Maribor, Maribor, Slovenia

**Keywords:** case report, neuromonitoring, angiography, indocyanine green, subarachnoid hemorrhage, arteriovenous malformation

## Abstract

In the surgical treatment of cerebral vascular malformations, e.g., aneurysms and arteriovenous malformations, the risk of ischemic complications is 6.7%, and a residual aneurysm is possible in 5.2% of these cases. Ischemic lesions can result in permanent neurological deficits, and a residual aneurysm can lead to the recurrence of the aneurysm in 2% of cases. In this article, we present five cases (two cases of ruptured aneurysms, two cases of non-ruptured aneurysms, and a case of arteriovenous malformation) in which we reduced the aforementioned risks with the use of intraoperative neuromonitoring and angiography. Intraoperative neuromonitoring (IONM) is used to measure motor and sensory-evoked potentials to detect brain hypoperfusion. Intraoperative angiography with the dye indocyanine green (ICG-A), which fluoresces in a vessel under a microscope after intravenous administration, helps to identify residual aneurysm sacs and distal blood flow. With the use of IONM and ICG-A, we identified abnormalities and adjusted our interventions and treatments. IONM and ICG-A can lead to a better outcome after surgical treatment of cerebral vascular abnormalities.

## Introduction

In the surgical treatment of vascular malformations, it is important that we achieve sufficient closure or removal of the malformation from circulation. On the other hand, we should keep normal blood vessels undamaged, and we should not make ischemic lesions with permanent neurological deficits. To improve the safety of procedures in vascular neurosurgery, intraoperative tools are being developed to lower the risk of complications. Among them are intraoperative neuromonitoring with monitoring of evoked potentials (EP), intraoperative angiography with fluorescent dye indocyanine green (ICG-A), micro-Doppler, and neuronavigation.

With IONM, we measure motor-evoked potentials (MEP) and sensory-evoked potentials (SEP). In measuring SEP, we monitor the sensory signal from the periphery to the sensory cortex. Used parameters include amplitude and latency N20 (the first negative peak of the cortical wave), N13 (subcortical peak), and central conduction time (CCT), representing the latency between N13 and N20 (1, 2). A drop in EP shows a conduction disorder that could be a consequence of an ischemic lesion following vasospasm or vessel closure. We considered a drop of the amplitude N20 by more than 50%, an increase in latency (N19/P24) by more than 10%, and/or an increase in CCT by more than 1 ms as relevant changes (3, 4). The slope measurement, which is defined as the relative slope amplitude and delay on every triggered potential peak in relation to the basal value, has an important role. This measurement has a significantly faster perceptible time than the so-called “peak-to-trough” method. The SEP method could discover a lesion in the area supplied by the medial cerebral artery, distal anterior cerebral artery, and basilar artery (3, 4). On the other hand, SEPs are susceptible to anesthesia maneuvers. Intravenous anesthetics, halogenated anesthetics, and nitric oxide act suppressively on SEPs, therefore weakening the amplitude and prolonging the delay of cortical response (N20), but not affecting the subcortical (N13) component (2).

During MEP measurements, we determine the functional integrity of the cortico-spinal tract (CST), in addition to the vascular territory of the cerebral vessels that perfuse it. Stimulation of the motor cortex can be achieved with electrodes placed at the C1 and C2 sites of the standardized international 10–20 system or with electronic meshes placed directly on the brain cortex. The criteria for a significant MEP change remain different. Some authors advocate the increase of the voltage threshold above 100 V as relevant (5–7), while others believe that only the complete loss of the MEP response is more predictive of cerebral ischemia (5, 8).

With the use of IONM techniques, we detect disturbances due to the placement of a temporary clip, due to the blockage of a smaller outflow artery when the clip is placed on the aneurysm, or we detect signs of vasospasm. ICG-A can be performed using a microscope that allows fluorescence. The dye is applied intravenously when the clip is placed on the neck of the aneurysm, and a video is recorded under fluorescence, which shows us how much contrast is still entering the aneurysm and whether any of the branches or perforating arteries are being closed by the clip. With the purchase of a new microscope and the inclusion of a neurologist on the team, we started using IONM and ICG-A for all cases of vascular malformations (ruptured and unruptured aneurysms and arteriovenous malformations – AVMs). We use a Kinevo microscope (Zeiss) and an Inomed neuromonitoring device.

## Case series

### First case

**Figure 1:**
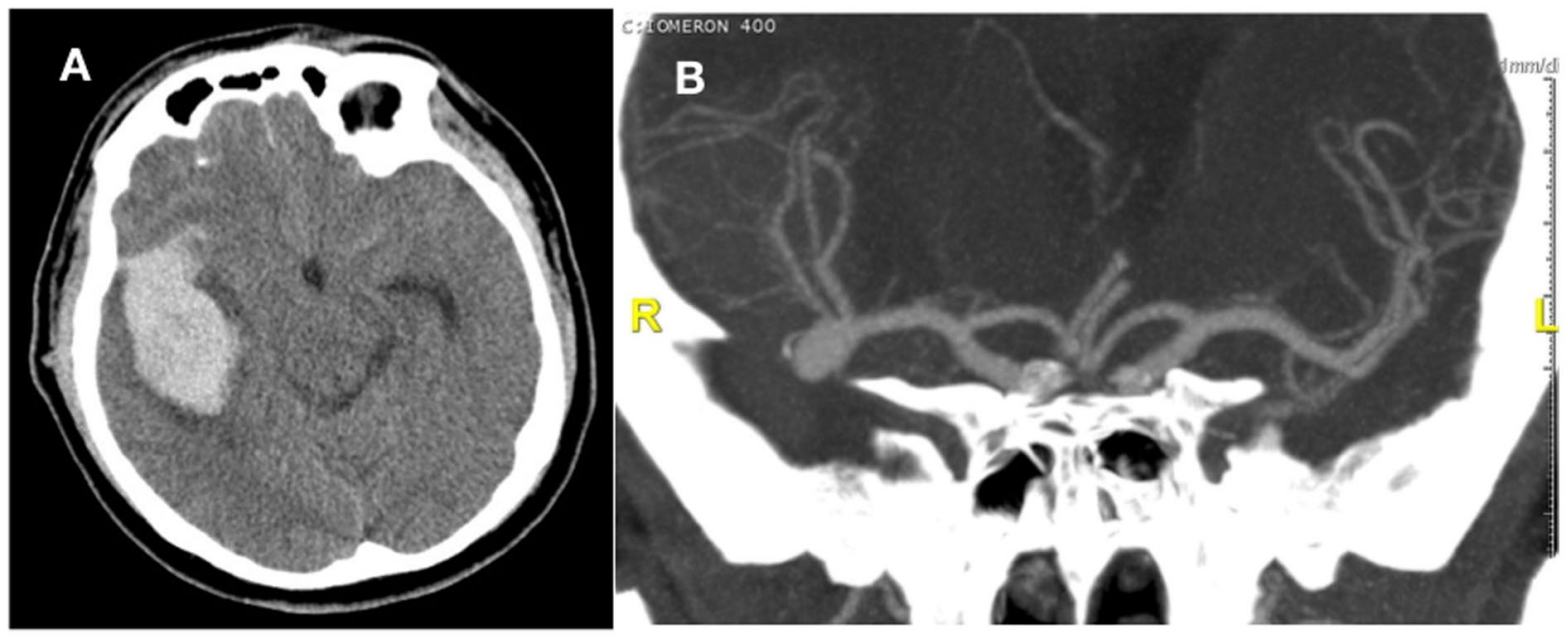
**(A)** CT scan with intracerebral hematoma. **(B)** CTA before surgery with an aneurysm on the trifurcation of the Middle cerebral artery (MCA).

A 50-year-old right-handed man was urgently admitted due to confusion and newly developing left-sided hemiparesis. He had arterial hypertension and a history of long-term smoking. On admission, his GCS was 14, his Hunt-Hess score was 2, he was confused, and in terms of neurological status, he had left-mouth corner droop, left-sided hemiparesis, and left-sided neglect. A CT scan of the head showed an intracerebral hematoma in the right parietal–temporal lobe measuring 6 × 2.6 cm and a subarachnoid hemorrhage (SAH) on the right side. CTA showed a 10 × 7 mm large aneurysm at the trifurcation of the right middle cerebral artery (MCA) (Figure 1).

The patient underwent urgent surgery, where a surgical clip was placed on the neck of the aneurysm through a right-sided pterional craniotomy. Hyperperfusion of the brain with systolic pressure above 130 mmHg was maintained during surgery, and we used neuromonitoring (specifically SEP). When the surgical clip was placed, the SEP fell to about 70% of the original value, and then when the arterial pressure was maintained at higher values, the SEP stopped falling and remained stable. MCA branches were free. This was followed by the evacuation of the hematoma from the temporal lobe and the insertion of the Intracranial Pressure (ICP) electrode. The patient was then transferred to the intensive care unit. There, he received standard therapy after SAH with nimodipine. The ICP values were at the upper limit; therefore, an EVD was inserted. A control CT scan of the head showed encephalomalacia with mild edema in the right temporal lobe. Later, he suffered a respiratory infection. After 15 days, sedation was finally discontinued, and the ICP and EVD were removed. After 18 days, he understood simple instructions and commands, and moderate to severe left-sided hemiparesis was present. Rehabilitation followed. After 19 months, the patient’s GOS (Glasgow outcome scale) score was 4, mRS (modified Rankin Scale) was 2, he had mild left-sided hemiparesis, partial left-sided visual field loss, lived at home, was independent in daily activities, was unable to work, and had symptomatic epilepsy. In this case, we reacted to the drop of SEP values in IONM with higher blood pressure and prevented a more severe ischemic lesion. ICG-A helped us confirm the position of the clip.

### Second case

**Figure 2:**
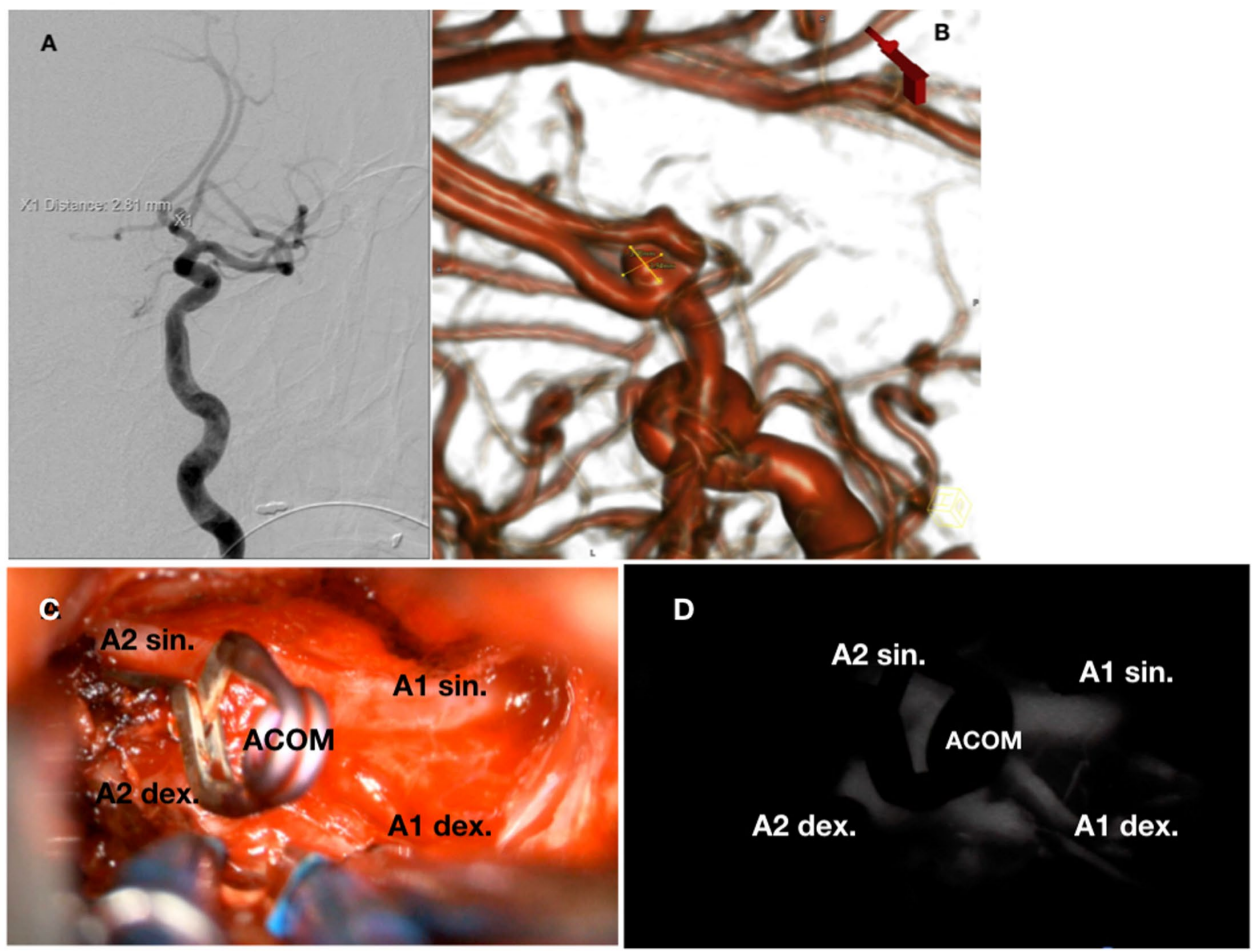
**(A)** DSA showing AComA aneurysm. **(B)** 3D DSA model of the aneurysm. **(C)** Intraoperative view of the clipped aneurysm and anterior cerebral arteries with A1, A2, and the communicating segment. **(D)** Same intraoperative view of vessels filled with indocyanine green dye.

A 42-year-old right-handed man with untreated arterial hypertension experienced a severe headache the day before admission and briefly lost consciousness. On admission, he had a GCS score of 15, Hunt-Hess I, and WFNS I. Head CT and CTA showed SAH and a 3 mm large aneurysm on the anterior communicating artery. DSA was performed, but endovascular embolization of the aneurysm was unsuccessful due to its wide base (Figure 2). The neuroradiologist positioned a coil into the aneurysm. Because of the wide neck of the aneurysm, the position of the coil in the aneurysm sack was unstable. As a result, the coil was removed, and it was decided against further attempts at endovascular embolization of the aneurysm.

The patient was treated surgically with a right pterional craniotomy and the placement of a surgical clip on the neck of the aneurysm. There was an intraoperative re-rupture of the aneurysm. After placing the clip, SEPs decreased (N20 amplitude to 60%, latency increased by 20%), but normalized as soon as systolic blood pressure rose above 140 mmHg and then remained the same. With ICG-A, the aneurysm was excluded after placing the clip, and both A1 and A2 segments were transitory (Figure 2).

An ICP electrode and EVD were inserted. The patient received nimodipine. Control CT the next day showed right frontal parasagittal hypodensity. TCD showed signs of vasospasm in the left MCA. A respiratory infection also appeared. ICP values were in the upper range. On the fifth day after the procedure, the patient died due to uncontrolled neurogenic pulmonary edema, mRS 6.

### Third case

**Figure 3:**
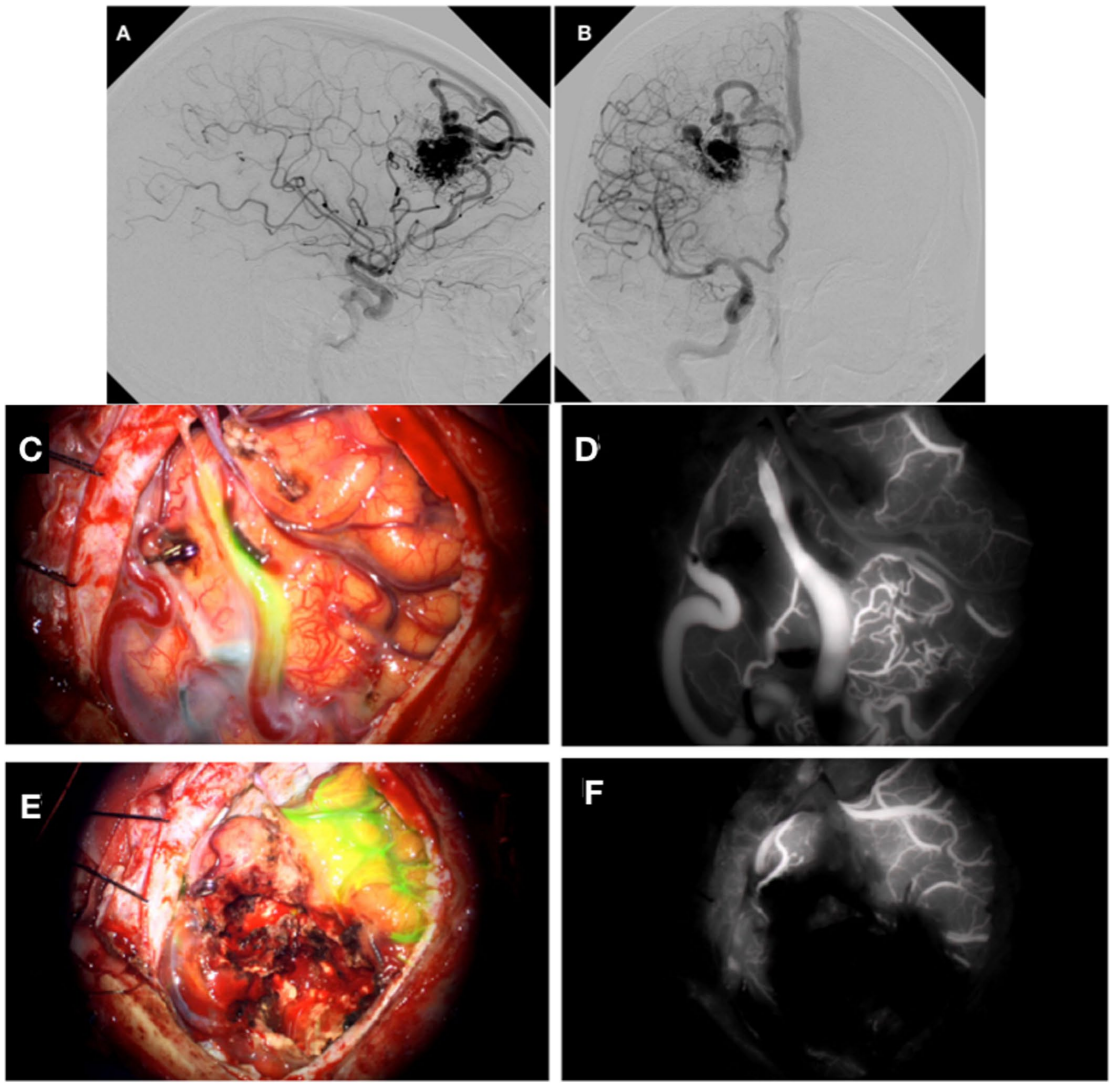
DSA demonstrating AVM from a lateral **(A)** and anterior **(B)** perspective. Cortical AVM after placement of a surgical clip on the feeding artery **(C)**. **(D)** Shows intraoperative angiography with indocyanine green dye. Image after removal of the AVM **(E)**. **(F)** Shows the intraoperative angiography with indocyanine green dye that did not show any coloration.

A 63-year-old right-handed woman with known arterial hypertension and rheumatoid arthritis suffered bleeding in the right frontal lobe due to an AVM (Spetzler-Martin grade 2, Lawton-Young scale grade 3). She recovered well and underwent endovascular embolization of the AVM. At the follow-up angiography one and a half years later, refilling and enlargement of the AVM were observed, and we decided to perform surgery. The feeding arteries for the AVM were from the right anterior cerebral artery (ACA) and smaller branches of the MCA. Drainage was into the superior sagittal sinus. The AVM measured 3.5 cm in diameter (Figure 3).

We performed the planned surgery with neuromonitoring. We approached the AVM through a right frontal craniotomy. With the help of ICG-A, we located the feeding artery and placed a surgical clip on it (Figure 3). After the removal of the AVM, we used ICG-A to rule out any remnants (Figure 3). Neurophysiological measurements of SEP and MEP remained unchanged during the procedure. The patient recovered well, and the control CT scan of the head showed postoperative changes without significant ischemic lesions. She has gradually recovered well, and after 6 months of follow-up, her GOS score was 5, mRS was 1 and she was without neurological deficits.

### Fourth case

**Figure 4:**
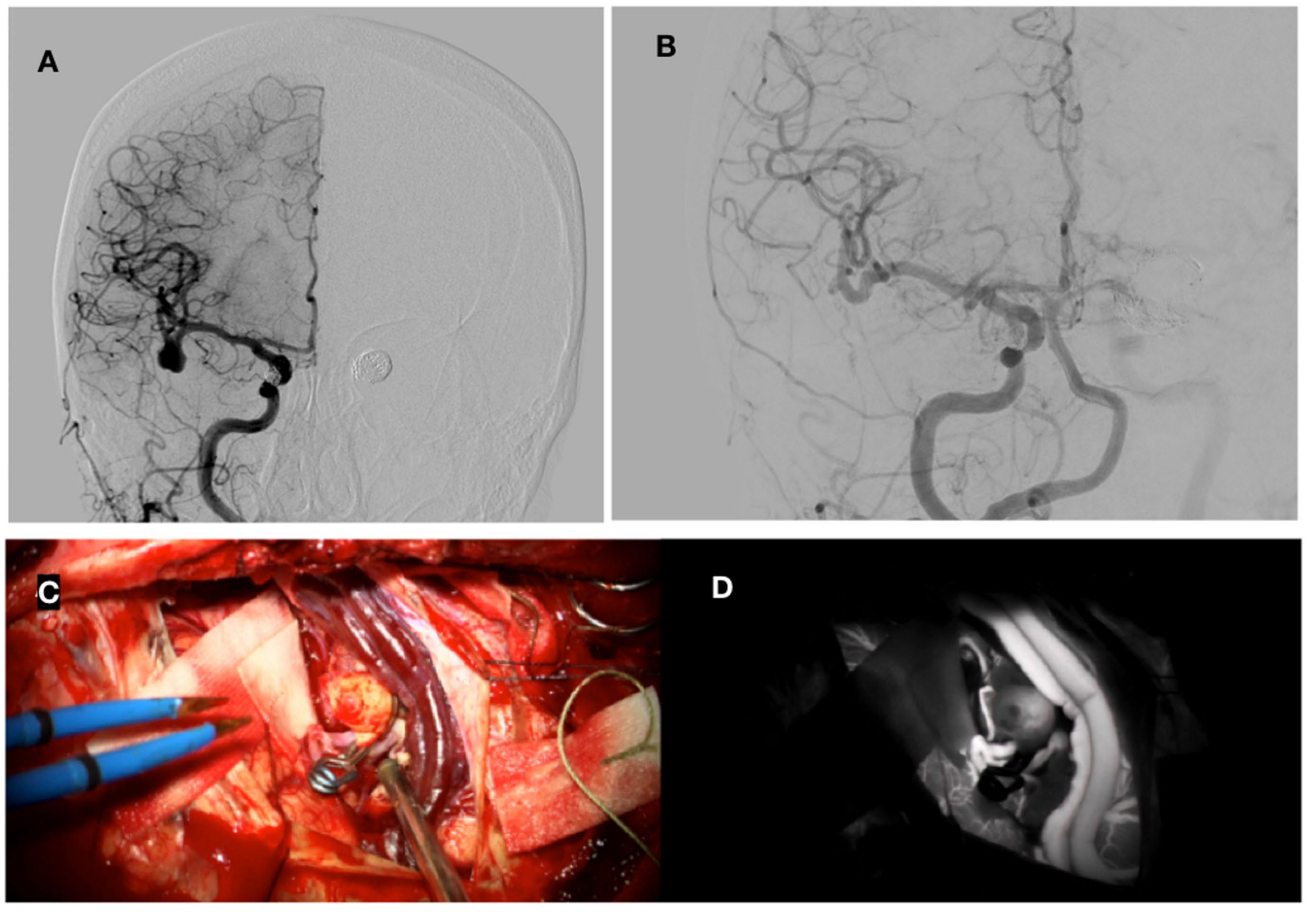
Preoperative DSA showing bisaccular aneurysm on MCA and coils after embolization of bilateral ACI **(A)**. Postoperative DSA with good closure of the aneurysm **(B)**. A surgical clip was placed on the aneurysm on MCA **(C)**. **(D)** Shows intraoperative angiography with indocyanine green dye.

A 72-year-old right-handed woman, a smoker, with no significant comorbidities, had multiple unruptured cerebral aneurysms. DSA showed aneurysms on the distal part of the left internal carotid artery (ACI), 15 mm in diameter, and a sister aneurysm on the distal part of the right ACI, 7 × 5 mm in size, in addition to one on the right MCA. The aneurysms on the distal part of the left and right ACIs were treated endovascularly with coils. After an unsuccessful attempt at endovascular embolization of the right MCA aneurysm (because of its wide neck and bisaccular shape), it was decided to treat it surgically. The aneurysm was at the bifurcation of the MCA, bisaccular, and measured 11 × 8 mm in size. Through a pterional craniotomy, a surgical clip was placed on the neck of the aneurysm at the bifurcation of the right MCA. SEPs decreased slightly upon placement of the surgical clip (N20 to 70%, latency lengthened by less than 15%) and then returned to normal as systemic blood pressure rose. ICG-A showed good exclusion of the artery from the circulation, and all branches of the MCA were free (Figure 4).

After the procedure, the patient initially woke up, but her GCS remained low with a value of 10–11, and the head CT showed two small intracerebral hematomas in both occipital lobes. Later control CT scans showed gradual resorption of the hematomas, her neurological condition improved, and she was discharged after 15 days; she remained independent in daily activities with minor difficulties in visual acuity. Angiography 6 months after the procedure showed the complete exclusion of the aneurysm after the procedure (Figure 4B); the GOS score was 5, and mRS was 1.

### Fifth case

**Figure 5:**
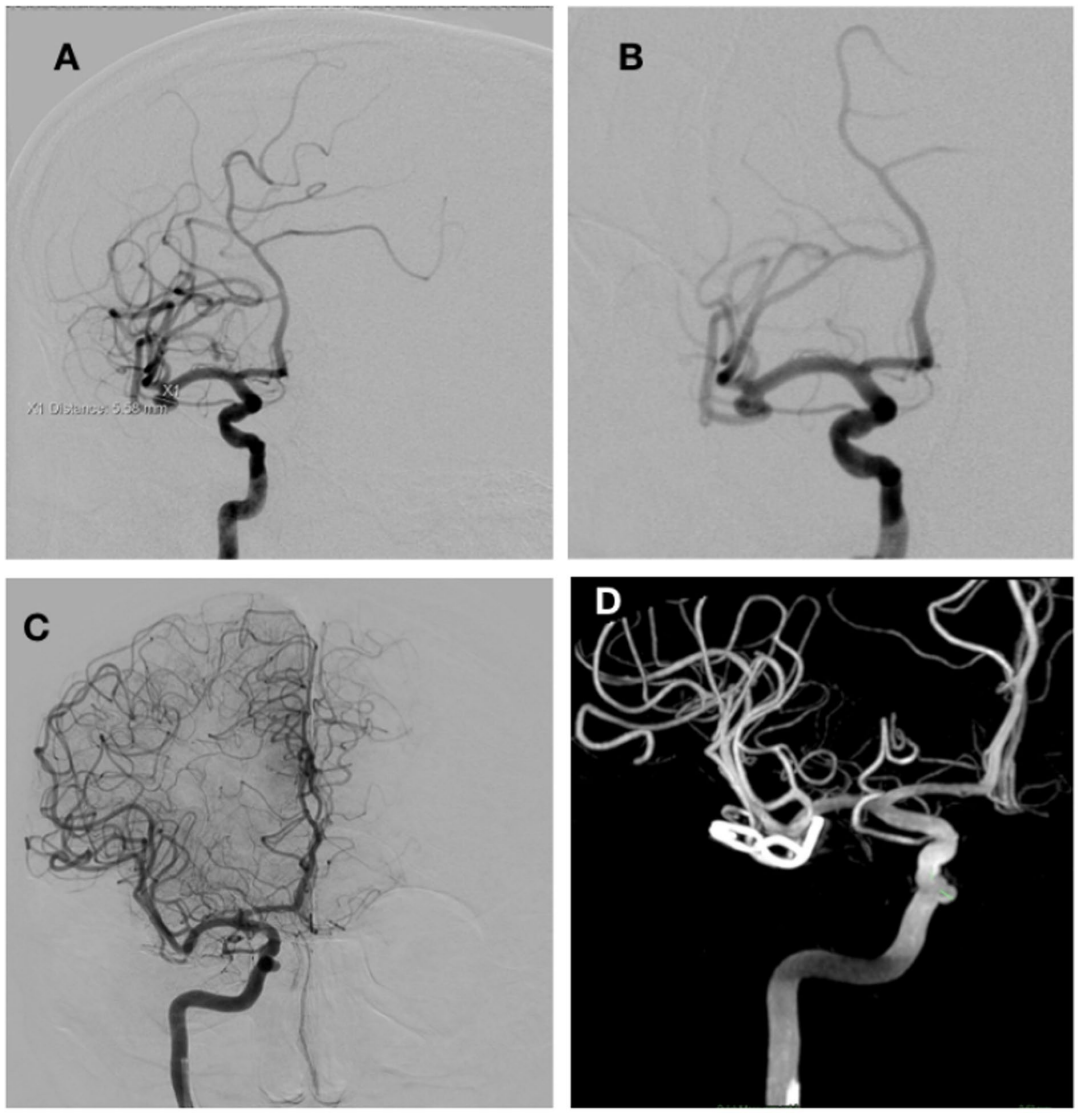
DSA before surgery showing an aneurysm on the MCA bifurcation, 5.4 × 4 mm in size **(A,B)**. **(C,D)** Show DSA images after surgery.

A 55-year-old right-handed man with multiple comorbidities (arterial hypertension, post-AMI condition, gout, sleep apnea) was diagnosed with an unruptured aneurysm of the right MCA. The aneurysm was at the bifurcation of the MCA, with a 3 mm wide neck measuring 5.5 × 4 mm (Figure 5). The patient had previously had two unsuccessful attempts at endovascular embolization. Through a right pterional craniotomy, two surgical clips were placed on the neck of the aneurysm. With IONM, we found a change in the slope of the SEP curve, which was followed by a prolongation of the CCT and a drop in amplitude, to which we immediately reacted with an increase in arterial pressure, while the SEP value initially stabilized and then normalized. ICG-A showed that there was no contrast leakage into the aneurysm and that all branches of the MCA were patent. The postoperative CT showed the condition after the procedure without significant bleeding or ischemia. A total of 15 days after the procedure, the patient was discharged; he displayed no neurological deficits and remained independent in his daily activities. At six months, GOS was 5, and mRS was 1.

## Discussion

The aim of surgical treatment of vascular malformations, especially in the case of incidentally found malformations with little or no symptoms, is to perform the procedures as safely as possible. Complications can be divided into vascular complications (leading to higher morbidity and mortality and permanent neurological deficits), damage to perforators, occlusion of the main artery, premature rupture of an aneurysm, thromboembolism, and non-vascular complications: local brain contusions, cranial nerve damage, excessive leak of cerebrospinal fluid, intracerebral hematoma, and infection (9). Ischemic complications account for 6.7% of all complications of unruptured aneurysms treated with surgical clips (4). Recurrence after surgery and vessel occlusion are the most important causes of complications after aneurysm surgery. In total after surgery, 5.2% of patients have a residual aneurysm, and 4.4% have a cerebral ischemic lesion (10). Even in emergency surgery, we want to perform the procedure as safely as possible and minimize any additional damage that a procedure can cause to an already affected patient. With this goal in mind, when the technical and human resources allowed, we started using IONM and ICG-A in surgeries for vascular malformations at our department.

A review of the literature shows that ICG-A has been used since 2003 (11, 12). Its advantages include a good evaluation of the complete exclusion of the aneurysm, the neck remnant, and the flow in the arteries and perforators. Disadvantages include a limited view of the operative field, the presence of clots, and intramural calcifications. The flow in the aneurysm after cliping is still present at 4–5% and ICG-A does not detect the remaining neck of less than 2 mm (9). In the retrospective studies presented, in 9% of cases, the surgical clip on the aneurysm was repositioned based on ICG-A, because either stenosis of the vessel or blockage of the perforators from the MCA was determined. These were not detected by the micro-Doppler. In 4.5% of cliped aneurysm an additional clip was used because after ICG-A the dye was still present in the aneurysm. In 9.1 % of cliped aneurysm the control DSA showed a neck remnant of less than 2 mm in size and in only one case a 6 mm remnant was found, which was not visible after ICG-A and required additional treatment later (5). ICG-A is a useful tool that leads to significant intraoperative changes in the surgical clip (15%), but a visible neck and aneurysm remnant are possible in 10% of patients. DSA examinations are still the most important examination for the evaluation of aneurysms, especially in the case of more complex ones (13).

SEPs are useful because they can be used to monitor the effects of anesthesia, surgical manipulation, and the placement of a temporary clip. In our cases, we mostly used SEP measurements because MEP measurements were not reliable due to the use of muscle relaxants and during emergency surgery. In most of our cases, except for AVM surgery, there was an immediate transient decrease in EP after placement of the surgical clip on the aneurysm. Therefore, we modified anesthetic therapy, increased systemic blood pressure, and administered intravenous nimodipine. In all cases, the decrease in potentials was quickly stopped, followed by a rise in EP and its normalization. In our cases, there was no neurological deterioration due to the procedure itself. In both emergency procedures, in the first case, the left-sided hemiparesis was due to a larger intracerebral hematoma around the aneurysm. In the second case, a vasospasm with an increase in ICP and uncontrollable neurogenic pulmonary edema occurred later. In the other planned surgeries, after a transitional period of recovery, which was complicated in one case by minor intracerebral hematomas and in two cases by epileptic seizures, a good recovery followed, without neurological deficits or the ability to function independently. Research also shows more variation in EP due to ruptured aneurysms. During aneurysm surgery, SEP change in 4% of unruptured cases, among them reversible changes led to ischemic dammage in 20% and irreversible changes led to ischemic damege in 80%. In 10% of ruptured cases, there was a change in SEP. A reversible change led to ischemia in 12% of patients and an irreversible change in 42% of them. Intraoperative SEP changes are thus more reliable in unruptured aneurysms. Irreversible changes led to ischemia in up to 80% of unruptured patients, but only in 42% of ruptured patients. Thus, the drop in SEP is not as fatal in ruptured aneurysms as in unruptured aneurysms (3).

A study comparing the use of SEP in unruptured aneurysm surgery found that ischemic complications occurred in 0.9% of the SEP group and 5.6% of the non-SEP group. At the same time, risk factors for ischemic complications include age over 62.5 years, size over 4.15 mm, temporary surgical clips, hyperlipidemia, history of ischemic stroke, and non-use of SEP. Caution is advised for MCA surgery in patients older than 62.5 years and with a history of ischemic stroke. SEPs have been found to be an effective and reliable tool to prevent ischemia during and after surgery (4). Significant changes in EP were found in 5.4% of 429 aneurysms, three of which had permanent motor neurological impairment (14). A reduction is considered significant when the EP amplitude is reduced by more than 50% (15). Intraoperative EP has a high specificity and negative predictive value. With a more accurate/sophisticated EP protocol, monitoring may prove to be a better indicator of a possible ischemic neurological deficit (15).

In the prediction of ischemia, SEPs have a positive predictive value of 30% and a negative predictive value of 94%. Sensitivity is 25%, and specificity is 95% (3). A meta-analysis found that the sensitivity and specificity for determining postoperative neurological deficit in SEP were 59 and 86%, in MEP were 81 and 90%, and in SEP + MEP were 92 and 88%, respectively. Thus, it is recommended to use a combination of SEP and MEP to predict postoperative strokes (1, 16). SEP proves to be highly specific after the placement of surgical clips. Patients with postoperative neurological deficits are seven times more likely to have changes on SEP as well. SEP monitoring can help initiate measures to prevent ischemic strokes after the placement of a surgical clip (1, 16). The problem with EPs is their low sensitivity (14). MEP mainly has better sensitivity and slightly better specificity. MEPs improve in accuracy and prediction when a direct strip electrode is used over the motor cortex (17). MEPs are superior to SEPs in detecting blood flow disturbances in perforating arteries, especially in subcortical ischemia due to lenticulostriate arteries and anterior choroidal arteries. MEPs indicate an ischemic event in the pyramidal tract. SEP + MEP is better than SEP alone for ACI and MCA. When we detect SEP/MEP disturbances, we stop the operation and administer nimodipine. Paresis that develops after the procedure despite normal MEPs during surgery is due to vasospasm and edema (10). Insufficient flow in the lenticulostriate (LSA) and cortical branches of the MCA that feed the cortico-spinal tract is detected by MEPs (18). SEPs are not reliable enough to detect reduced blood flow through the MCA and LSA branches (18).

The cooperation of an anesthesiologist and a neurophysiologist is important. The type of anesthetics, the use of neuromuscular blockers, and the selection of diagnostic criteria for a significant change in cerebral blood flow during surgery affect the diagnostic accuracy of EP techniques in the prediction of postoperative neurological outcomes (17). With changes in SEP/MEP (amplitude drop, latency increase, or loss of curve), we should react by releasing the surgical clip, releasing the temporary clip, or local application of nimodipine (10). Compared to ICG-A, SEP/MEP measurements are more effective in identifying ischemic defects in anterior aneurysms. ICG-A is effective in ruling out the risk of residual aneurysms, leading to 2.9% of recurrent aneurysms (10). The causes of flow disturbances in the perforating arteries include stenosis of the main artery due to a temporary surgical clip or occlusion of the main artery and direct damage to the vessel wall (10).

In contrast to the majority of research, the study by Greve et al. concluded that the introduction of SEP/MEP in operations for unruptured cerebral aneurysms did not contribute to significantly better results. Therefore, they summarize that IONM is not absolutely necessary in all neurovascular interventions (19). It is important that IONM be performed until the end of the procedure. An example of the rotation of an aneurysmal surgical clip is described, which occurred after the relaxation of brain retraction and reduced the flow in the branches of the artery next to the aneurysm (20). The theory of pressure increase after compression of the aneurysm with the clip is also mentioned by Yamada et al. (21). In three cases where they had a normal MEP, they had a late ischemic response, which may be the result of hypotension or vasospasm after the end of anesthesia (21). MEP disturbances occur in somewhere between 5.4 and 25% of aneurysm surgeries. Changes to the MEP should, as far as possible, be corrected in less than 5 min. Permanent loss of MEP is associated with irreversible progressive ischemia and paresis (21).

## Conclusion

The use of IONM and ICG-A can increase the safety of surgery and contribute to a better final outcome. In the future, even greater efficiency will require the introduction of standardized protocols for both surgical and anesthetic preparation. This will optimize preoperative preparation, expedite urgent procedures, and allow for comparison and evaluation.

## Data availability statement

The raw data supporting the conclusions of this article will be made available by the authors, without undue reservation.

## Ethics statement

The studies involving human participants were reviewed and approved by the Medical Ethics Commission of the University Medical Centre Maribor. The patients/participants provided their written informed consent to participate in this study. Written informed consent was obtained from the individual(s) for the publication of any potentially identifiable images or data included in this article.

## Author contributions

TŠ, NK, and JR contributed to the conception and design of the study. TŠ and JR performed surgeries on patients, analyzed cases, and wrote sections of the manuscript. NK performed intraoperative neuromonitoring, analyzed cases, and wrote sections of the manuscript. All authors contributed to the manuscript revision and read and approved the submitted version.
